# Early Occupational Therapy intervention to prevent contractures in person with Dementia – A Case Series

**DOI:** 10.1002/alz70858_106712

**Published:** 2025-12-25

**Authors:** Sridharan K, Priya Treesa Thomas, Srinithya G, Faheem Arshad, Suvarna Alladi

**Affiliations:** ^1^ National INstitute of Mental Health And Neuro Sciences, Bengaluru, karnataka, India; ^2^ National Institute of Mental Health and Neurosciences [NIMHANS], Bengaluru, Karnataka, India; ^3^ National Institute of Mental Health and Neurosciences, Bengaluru, Karnataka, India

## Abstract

**Background:**

Joint contractures are very common in persons with dementia who are confined to bed in the terminal stage, which will lead to difficulty in transferring the patients from one place to another, difficulty in maintaining the posture while feeding all those will increase the caregiver burden, and also affects the patient's quality of life. The aim of the study is to prevent contractures by applying earlier occupational therapy interventions.

**Method:**

Patients diagnosed with Dementia who are registered under the Neuropalliative care project at NIMHANS a tertiary care center for neurological conditions were consecutively recruited. The study follows a case series design. Informed consent was obtained from all the participants. All the four participants received routine Occupational Therapy interventions aimed at preventing contractures. The interventions were tailored to the specific needs of each participant and the intervention included: Passive Range of Motion, Assistive device prescription like an Elbow immobilizer, Resting Hand Splint, Knee immobilizer, Ankle Foot Orthosis, Positioning strategies, and Caregiver education regarding long‐term care management. The impact of the interventions was routinely assessed throughout follow‐up visits.

**Result:**

The recruited participants who were confined to wheelchair‐bound stage received an earlier intervention through home care visit. These interventions continued throughout the patient's life (up to two years in some cases) and are ongoing for those still alive. It was identified that Patients who consistently follow the occupational therapy early intervention techniques helped to avoid developing contractures, even in the terminal stage. In contrast, patients whose families did not adhere to the prescribed techniques experienced severe contractures, leading to increased caregiver burden. A detailed analysis will be presented.

**Conclusion:**

This study highlights that occupational therapy's early intervention plays a major role in contracture prevention, reducing caregiver strain, and enhancing patient well‐being.

